# Causal effect estimation on restricted mean survival time under case-cohort design via propensity score stratification

**DOI:** 10.1007/s10985-025-09667-w

**Published:** 2025-08-18

**Authors:** Wei-En Lu, Ai Ni

**Affiliations:** Division of Biostatistics, College of Public Health,Ohio State University, Columbus, USA

**Keywords:** ARIC study, Causal effect estimation, Propensity score stratification, Restricted mean survival time, Stratified case-cohort design, Survival analysis

## Abstract

In large observational studies with survival outcome and low event rates, the case-cohort design is commonly used to reduce the cost associated with covariate measurement. The restricted mean survival time (RMST) difference has been increasingly used as an alternative to hazard ratio when estimating the causal effect on survival outcomes. We investigate the estimation of marginal causal effect on RMST under the stratified case-cohort design while adjusting for measured confounders through propensity score stratification. The asymptotic normality of the estimator is established, and its variance formula is derived. Simulation studies are performed to evaluate the finite sample performance of the proposed method compared to several alternative methods. Finally, we apply the proposed method to the Atherosclerosis Risk in Communities study to estimate the marginal causal effect of high-sensitivity C-reactive protein level on coronary heart disease-free survival.

## Introduction

### Case-cohort design

The case-cohort design is a cost-effective approach used in large observational studies to investigate risk factors for survival outcomes (Prentice 1986). In this context, the “case" refers to subjects who develop the event during the study period. This design involves randomly selecting a subcohort from the full cohort, with unselected cases added to the subcohort to form the case-cohort. Covariates are only measured in the case-cohort. It is particularly useful when collecting covariate history for all individuals is impractical due to cost constraints, especially in large studies. The case-cohort design is especially advantageous when event rates are low, reducing data collection costs and time with minimal efficiency loss (Prentice 1986).

In practical applications, some covariates that are readily available for the full cohort, such as age, weight, and sex, may be correlated with the more expensive covariates. To make use of these fully observed covariates, a stratified case-cohort design was proposed, where the full cohort is stratified based on these covariates, and case-cohort sampling is carried out within each stratum (Borgan et al. 2000).

Analyzing case-cohort studies involves addressing the specific challenges rising from the case-cohort sampling as the ratio of case to non-case in the case-cohort is not representative of the full cohort. One class of methods focuses on the construction of a pseudo-likelihood function in which the risk sets are modified by certain weights to account for the case-cohort study design (Prentice 1986; Self and Prentice 1988; Kalbfleisch and Lawless 1988; Borgan et al. 2000; Kulich and Lin 2004). Another perspective views case-cohort studies as a missing-data problem, since subjects outside the subcohort have unobserved covariates, with the observation process controlled by study design. Marti and Chavance (2011) suggested using multiple imputation as an alternative to weighted analysis to handle this missing covariate information.

### Causal inference in observational survival data

In large observational studies, it is often important to establish the causal relationship between a treatment or exposure and a survival outcome. There are many challenges to causal inference in observational survival data. First, standard statistical analysis methods are not sufficient to account for censoring associated with survival data. Second, while most studies measure the association with the hazard ratio (HR), it is not an appropriate measure of the marginal causal effect due to its conditional nature that induces selection bias (Hernán et al. 2004; Hernán 2010; Ni et al. 2021). Third, the Cox proportional hazards model is often used to estimate the HR but the proportional hazards assumption may not always be satisfied. Finally, observational survival data may suffer from confounding effects, as treatment groups often differ systematically in the distribution of the baseline covariates. Propensity score-based methods are commonly used to adjust for confounding effects. These methods include propensity score stratification (Rosenbaum and Rubin 1984; D’Agostino 1998; Cook and Goldman 1989; Lunceford and Davidian 2004; Austin 2011), matching (Rosenbaum and Rubin 1983; D’Agostino 1998; Rosenbaum et al. 2010; Stuart 2010; Zhao and Lu 2023), and weighting (Hahn 1998; Hirano and Imbens 2001; Hirano et al. 2003; Lunceford and Davidian 2004; Imbens 2004). In this paper, we employ propensity score stratification for confounding adjustment.

### Restricted mean survival time (RMST)

The difference in restricted mean survival time (RMST) is an alternative effect measure of the survival outcome to the HR. RMST, denoted as $\mu (\tau )$, is the expected survival time during the first $\tau$ follow-up period and is equivalent to the area under the survival curve up to $\tau$ (Irwin 1949; Zhao and Tsiatis 1997; Andersen et al. 2004). Formally,

$$\begin{aligned} \mu (\tau ) = \int _0^\tau S(t) dt \end{aligned}$$

where *S*(*t*) is the survival function. The truncation time point $\tau$ is typically specified *a priori* based on subject knowledge. The treatment effect can be estimated as the difference in RMST between the treated and control arms. RMST difference is easy to interpret, collapsible, free of selection bias, and does not rely on the proportional hazards assumption (Ni et al. 2021; Lin et al. 2023). RMST does not suffer from selection bias because it is not a conditional quantity but rather an integration over follow-up time $\tau$. RMST can be estimated nonparametrically by calculating the area under the Kaplan-Meier estimate of the survival function.

As mentioned in the previous section, propensity score stratification can be applied to remove confounding bias in observational studies. By stratifying on the propensity score, the covariate distribution between treated and control becomes similar within each propensity score stratum. Ni et al. (2021) proposed to use propensity score stratification to remove observed confounding bias and to estimate the RMST difference between treatment groups as a marginal causal effect measure. In this paper, we extend their method to the stratified case-cohort design so that causal effects can be estimated for large observational studies with survival outcome that utilize such a sampling design.

### Motivating example (ARIC study)

Coronary heart disease (CHD) is one of the leading causes of disability and death in the United States. High-sensitivity C-reactive protein (hs-CRP) is often associated with the risk of CHD (Katan and Luft 2018; Virani et al. 2021; Kochanek 2014). However, the causal relationship remains uncertain (Casas et al. 2008; Hingorani et al. 2009). The Atherosclerosis Risk in Communities (ARIC) study, an observational investigation involving 15,792 middle-aged US adults over several decades, aims to study the development of cardiovascular events and health-related issues (Ballantyne et al. 2004). An aspect of the ARIC study is to examine the association between inflammatory biomarkers such as hs-CRP and the risk of coronary heart disease. Due to the limited collection of plasma samples, a stratified case-cohort design was employed, stratified by age, sex, and race, with hs-CRP measured only in the selected case-cohort sample.

## Methods

### Notations and assumptions

In an observational time-to-event study, suppose the research interest is in the causal effect of a binary treatment. Let *A* be the treatment indicator where $A=0$ denotes control arm and $A=1$ denotes treatment arm. Let *X* be a *p*-dimensional baseline covariate vector. Let $T^A$ denote the potential event time under treatment *A* and $S^A(t)$ be its survival function. With truncation time point $\tau$, $Z^A = \min (T^A, \tau )$ is the potential restricted event time. Let *C* be the random censoring time and $T=AT^1 + (1-A)T^0$ be the observed event time. The observable quantities are *U*, $\tilde{Z}$, and $\delta _{\tilde{Z}}$, where $U = \min (T,C)$ is the unrestricted observation time, $\tilde{Z} = \min (Z,C)$ is the restricted observation time, and $\delta _{\tilde{Z}} = I(Z<C)$ is the event indicator. Let $N(t) = I(T\le t)$ be the counting process and $Y(t) = I(T \ge t)$ be the at risk process. Under treatment assignment *A*, the potential restricted mean survival time is $\mu ^A(\tau ) = \int _0^\tau S^A(t)dt$. Then the marginal causal effect of the treatment $\nu$ is defined as

$$\begin{aligned} \nu = \mu ^1(\tau ) - \mu ^0(\tau ) = \int _0^\tau \big [ S^1(t) - S^0(t)\big ]dt. \end{aligned}$$

We require the following assumptions to estimate causal effects on RMST with propensity score stratification.

#### Assumption 1

Stable Unit Assumption (SUTVA): The potential survival times for one individual in the population do not vary with the treatment assigned to others, and there are no different versions of the specified treatment level.

#### Assumption 2

Strongly Ignorable Treatment Assignment Assumption (SITAA): $(T^0, T^1) \perp A \mid X$ and $0< P(A=1\mid X) < 1$.

#### Assumption 3

Independent censoring: The censoring variable, *C*, is independent of $T^A$ given treatment *A*.

#### Assumption 4

The truncation time point $\tau$ lies between the minimum event time and the maximum follow-up time (event or censored) of each treatment group in each propensity score stratum in the case-cohort sample.

Note that Assumption 3 implies that $C \perp Z^A \mid A$ since $\tau$ is a constant, while Assumption 4 ensures asymptotically unbiased RMST estimates and nonzero variance estimates. In a stratified case-cohort study with *B* strata, the subcohort sampling probability in stratum *b* ($b=1,...,B$) is denoted as $\alpha _{b}$, and we define $\xi _{bi}$ as 1 if subject *i* in stratum *b* is included in the subcohort and 0 otherwise where $i=1,\dots ,n_b$ and $n_b$ is the size of stratum *b* in the subcohort. The case-cohort comprises all subjects from the subcohort and all cases outside the subcohort. In this paper, we use Bernoulli sampling to obtain the subcohort, where all $\xi$’s are independent. Some or all variables in *X* are observed only for members of the case cohort.

### Propensity score stratified RMST estimator under stratified case-cohort design

We propose, under a stratified case-cohort design, to use the RMST difference as the measure of marginal causal effect and to use propensity score stratification to remove confounding bias. The proposed method involves the following steps: stratified case-cohort sampling, propensity score estimation, propensity score stratification, and treatment effect estimation.

#### Stratified case-cohort sampling

Assume that there are *N* subjects in the full cohort sample. To take a stratified case-cohort sample, one first takes a random subcohort by Bernoulli sampling from each of the *B* strata of the original cohort stratified by some baseline covariates and then adds cases outside the subcohort to form a stratified case-cohort sample. When RMST is used as the outcome, the definition of the case slightly deviates from the typical one for survival data. In addition to traditional cases where the event time is observed during the follow-up period, subjects who are followed to $$\tau $$ without an event or censoring are also counted as cases because they contribute complete information to the study period $$[0, \tau ]$$. Thus, the case-cohort design is more efficient if these subjects are included as cases. Expensive covariates, which may include treatment, are measured only in the case-cohort sample. Denote the full cohort size of stratum *k* as $$N_k$$ ($$k=1,...,K)$$, case-cohort size of stratum *k* as $$n_k$$, and the total case-cohort size as $$n=\sum _{k=1}^K n_k$$. We use Estimator II in Borgan et al. (2000) to estimate the risk set at each event time, where the sampling weight for subject *i* in the case-cohort stratum *b* is $$\rho _{bi} = \delta _{Z,b_i} + (1-\delta _{Z,b_i})\frac{\xi _{bi}}{\alpha _{b}}$$ where $$\alpha _b$$ is the sampling probability of the subcohort in stratum *b*, $$\delta _{Z,b_i} = I(\tilde{Z}_{bi} < C_{bi})$$ is the case indicator based on the new definition of case and $$\xi _{bi}$$ is the subcohort indicator.

#### Propensity score estimation and stratification

The propensity score can be estimated based on the case-cohort sample by fitting a weighted logistic regression on the treatment variable *A* weighted by the case-cohort weight $\rho$ to account for the case-cohort sampling. Assume that for subject *i* in the case-cohort sample, we observe a binary treatment variable $A_i$ following a Bernoulli distribution with parameter $p_{i0}=e^{\varvec{x_i}'\beta _0}(1+e^{\varvec{x_i}'\beta _0})^{-1}$ in a logistic regression model, where $\varvec{x_i}$ is a *p*-dimensional covariate vector. Under a case-cohort design, *A* and $\varvec{x}$ are observed only in the case-cohort sample. Therefore, in the logistic regression, each subject is weighted by their case-cohort sampling weight $\rho _i$ to account for the sampling design. The regression coefficient is estimated by maximizing the log-likelihood of the weighted logistic regression $l^w(\beta \mid A) = \sum _{i=1}^N A_i \rho _i \varvec{x}_i' \beta - \sum _{i=1}^N \rho _i \log (1+e^{\varvec{x}_i'\beta })$. Denote the maximizer of the log-likelihood as $\hat{\beta }^w$ and define the estimated propensity score for subject *i* as $\hat{p}_i=e^{\varvec{x}_i'\hat{\beta }^w}(1+e^{\varvec{x}_i'\hat{\beta }^w})^{-1}$, $i=1,...,n$.

##### Proposition 1

A weighted logistic regression produces consistent estimates of the propensity score, i.e. $\hat{p}_i \xrightarrow {p} p_{i0}$ for $i=1,...,n$.

The proof of Proposition 1 follows by using the law of total expectations on the score function of the weighted logistic regression and is included in Appendix 1.1. Once the propensity score is estimated for each subject in the case-cohort, the case-cohort sample will be stratified by the propensity score into *K* strata of equal sizes. It is recommended to stratify the sample into five to ten strata (Rosenbaum and Rubin 1984). In this paper, seven equal-sized strata are chosen based on percentiles of the propensity score distribution. After stratifying by propensity score, the marginal treatment effect is estimated by taking the weighted average of the propensity score stratum-specific RMST difference between the treated and control.

#### RMST estimation under stratified case-cohort design

In a stratified case-cohort sample, let $t_l$ ($l=1,...,L$) be the *L* unique event times. We propose to estimate the survival function using a weighted Kaplan-Meier estimator

$$\begin{aligned} \hat{S}_{cc}(t)&= \prod _{0 \le t_l \le t}\Bigg ( 1- \frac{\sum _{i=1}^N dN_i(t_l)}{\sum _{j=1}^NY_j(t_l)\rho _j}\Bigg ), \end{aligned}$$

where $\rho _i$ is the sampling weight for subject *i*. The RMST can be estimated by $\hat{\mu }_{cc}(\tau ) = \int _0^\tau \hat{S}_{cc}(t)dt$.

##### Theorem 1

Under Assumptions 3, 4, and a stratified case-cohort design with *B* strata and *N* subjects in the full cohort sample, $\sqrt{N}(\hat{\mu }_{cc}(\tau ) - \mu (\tau ))$ converges to a normal distribution with mean zero and variance $\int _0^\tau \overline{\mu }^2(\tau ,u)d\textrm{Var}_s(u)$ as $N\rightarrow \infty$, where

$$\begin{aligned} \overline{\mu }(\tau ,u) =&\mu (\tau ) - \mu (u) = \int _u^\tau S(v)dv, \\ \textrm{Var}_s(u) =&\int _0^u \pi ^{-1}(v)d\varLambda _0(v) + \\&\Big (\sum _{b=1}^Bp_b\frac{1-\alpha _b}{\alpha _b}\Big ) \textrm{E}\Big [\int _0^u Y(v)(1-\delta )\pi ^{-1}(v)d\varLambda _0(v) \Big ]^2, \\ \pi (u) =&\lim _{n\rightarrow \infty } \frac{\sum _{j=1}^N Y_j(u)}{N} = \textrm{E}[Y(u)], \end{aligned}$$

$p_b$ is the proportion of subjects in case-cohort stratum *b*, and $\varLambda _0(\nu )$ is the true cumulative hazard function.

The proof of Theorem 1 is provided in Appendix 1.2.

Note that Theorem 1 pertains to a general case-cohort sample without treatment or propensity score stratum. Under a case-cohort design, the selected non-cases constitute a simple random sample of the non-cases in the full cohort. The selection probability is a design parameter independent of any outcome or covariates. Therefore, in each treatment group within each propensity score stratum, the selected non-cases also constitute a simple random sample of the non-cases in the full cohort for the given treatment group within the given propensity score stratum. Moreover, cases in the full cohort are always selected. Therefore, cases in the full cohort for any given treatment group within any given propensity score stratum are also always selected. Thus, the selected sample in any given treatment group within a propensity score stratum can also be deemed as a case-cohort sample for that specific treatment group and propensity score stratum. Therefore, Theorem 1 can be applied to each propensity score stratum in each treatment group.

To estimate the variance of $\hat{\mu }_{cc}(\tau )$, $\textrm{Var}_s(u)$ can be estimated by

$$\begin{aligned} \widehat{\textrm{Var}}_s(u) =&N \Bigg \{ \sum _{0<t_l<u} \frac{1}{(\sum _{j=1}^N Y_j(t_l)\rho _j)^2} + \\&\sum _{i=1}^N \frac{1-\alpha _i}{\alpha _i}\rho _i\Big (\sum _{0<t_l<u} \frac{Y_i(t_l)}{(\sum _{j=1}^N Y_j(t_l)\rho _j)^2}\Big ) ^2\Bigg \}. \end{aligned}$$

Thus, the variance of $\hat{\mu }_{cc}(\tau )$ can be estimated by

1 $$\begin{aligned} \widehat{\textrm{Var}}\big (\hat{\mu }_{cc}(\tau )\big ) = \frac{1}{N}\sum _{l=1}^{n_e}\Big (\int _{t_l}^\tau \hat{S}_{cc}(u) du \Big )^2 \Big [\widehat{\textrm{Var}}_s(t_l) - \widehat{\textrm{Var}}_s(t_{l-1}) \Big ], \end{aligned}$$

where $n_e$ is the total number of event times.

Under Assumptions 1 and 2, the treatment effect specific to a propensity score stratum is estimated by taking the difference of the estimated RMST within each propensity score stratum between the treated and control groups. Define the stratum-specific causal effect of treatment as $\nu _k(\tau ) = \mu ^1_k(\tau ) - \mu ^0_k(\tau ) = \int _0^\tau \big [ S_{k}^1(t) - S_{k}^0(t) \big ]dt$ where $\mu ^1_k(\tau )$ and $\mu ^0_k(\tau )$ are the potential RMST of the treated and control groups within stratum *k*, respectively, and $S_{k}^1(t)$ and $S_{k}^0(t)$ are the potential survival functions of the treated and control groups within stratum *k*, respectively. Conditional on the propensity score stratum *k*, the strong ignorability assumption allows us to estimate $\nu _k(\tau )$ with $\hat{\nu }_k(\tau ) = \hat{\mu }_{k1}(\tau ) - \hat{\mu }_{k0}(\tau ) = \int _0^\tau \big [ \hat{S}_{cc,k1}(t) - \hat{S}_{cc,k0}(t) \big ]dt$ where $\hat{S}_{cc,k1}(t)$ and $\hat{S}_{cc,k0}(t)$ are the weighted Kaplan-Meier estimates of the survival function for the treated and control groups in stratum *k*, respectively.

The marginal treatment effect is estimated by a weighted average of stratum-specific treatment effects: $\hat{\nu }(\tau ) = \sum _{k=1}^K w_k \hat{\nu }_k(\tau )$. The weights $w_k$ can be chosen to estimate the average treatment effect (ATE) or the average treatment effect for the treated (ATT). For ATE, $w_k = \sum _{i=1}^{N_k} \rho _{ki}/\sum _{k=1}^K\sum _{i=1}^{N_k} \rho _{ki}$ where $\rho _{ki}$ is the case-cohort weight of subject *i* in the propensity score stratum *k*. For ATT, $w_{k} = \sum _{i=1}^{N_k} I(A_{ki}=1)\rho _{ki}/\sum _{k=1}^K \sum _{i=1}^{N_k}I(A_{ki}=1)\rho _{ki}$, and $I(A_{ki}=1)$ is the treatment indicator of subject *i* in the stratum *k*. Define $\nu (\tau )=\sum _{k=1}^K \textrm{E}(w_k) \nu _k(\tau )$.

##### Corollary 1

With stratum-specific weight $w_k$, the estimate of the marginal treatment effect $\hat{\nu }(\tau ) = \sum _{k=1}^K w_k \hat{\nu }_k(\tau )$ is asymptotically normal with mean $\nu (\tau )$ and variance $\sum _{k=1}^K [\textrm{E}(w_k)]^2 [\textrm{Var}(\hat{\mu }_{k1}(\tau )) + \textrm{Var}(\hat{\mu }_{k0}(\tau ))]$ as $n_k$ approaches infinity for all $k=1,...,K$, where $\textrm{Var}(\hat{\mu }_{k1}(\tau ))$ and $\textrm{Var}(\hat{\mu }_{k0}(\tau ))$ are estimated by (1).

The proof of Corollary 1 is in Appendix 1.3. Under finite sample sizes, the distributions of covariates are only roughly equal within each propensity score stratum. This results in residual confounding in each propensity score stratum. Thus, the propensity score stratified estimators of both ATE and ATT are only approximately unbiased. The degree of bias depends on the magnitude of the residual confounding in the propensity score strata. If *K* approaches infinity with *N*, the distributions of the covariates will become exactly the same within each propensity score stratum. Therefore, the residual confounding approach zero and the bias of the propensity score stratified estimators converges to zero.

Since the unstratified case-cohort design is a special case of the stratified case-cohort design with a strata number of one, the consistency and asymptotic normality of the proposed estimators remain the same for unstratified case-cohort design.

## Simulation study

### Data generation

We generate six baseline covariates consisting of three continuous covariates, $X_1$, $X_2$, $X_3$, and three binary covariates, $X_4$, $X_5$, $X_6$. First, $X_1$, $X_2$, $X^*_4$, $X^*_5$ are generated from a multivariate normal distribution with mean of one, variance of 2, and pairwise covariance of 0.5. The two binary covariates, $X_4$ and $X_5$, are then obtained by dichotomizing $X^*_4$ and $X^*_5$ at one. $X_3$ is defined as $X_3 = 0.2 X_1 + 0.1 X_4 + 0.2 X_1 X_4 + \epsilon$ and $X_6$ is defined by dichotomizing $X^*_6 = 0.2 X_1 - 0.4 X_4 - 0.9 X_1 X_4 + \epsilon ^*$ at its median where $\epsilon$ and $\epsilon ^*$ are normally distributed with mean zero and variance of 0.5. The treatment indicator, *A*, is generated from a Bernoulli distribution where the treatment assignment probability, $P(A=1)$, is specified as $\textrm{logit}[P(A=1)] = 0.85 + 0.4 X_1 -0.4 X_2 + 0.3 X_3 + 0.4 X_4 - 0.4 X_5 - 0.4 X_6$. In this setting, approximately $60 \%$ of the subjects receive treatment.

Independent failure times are generated from a proportional hazards model. Potential survival times $T^A$ are generated from an exponential distribution with hazard $\lambda (t) = \exp (0.5 + \beta _A A -0.5 X_1 + 0.5 X_2 + 0.4 X_3 -0.5 X_4 + 0.5 X_5 - 0.4 X_6)$. The censoring variable *C* is generated from an exponential distribution with hazard $\theta (t) = \exp (\gamma -0.5A)$ and $\gamma$ is specified to achieve the desired censoring rates. The true restricted event time *Z*, the unrestricted observation time *U*, and the restricted observation time $\tilde{Z}$ are generated according to their definition. In this simulation, $\tau$ is chosen to be the empirical 85th percentile of the unrestricted observation time *U* in a simulated dataset with an extremely large sample size, and $\gamma$ is specified so that $5\%$ of the subjects experience an event before $\tau$ and an additional $15\%$ of subjects who survive up to or beyond $\tau$. Based on the modified definition of case (see Section 2.2.1), there are a total of $20\%$ cases in the full cohort, corresponding to an 80% censoring rate.

After generating the full cohort, a stratified case-cohort sample is drawn from it. The full cohort is stratified by the binary variable $X_4$ and stratum-specific case-cohort sampling probabilities are determined to achieve the desired non-case to case ratios in each stratum. Covariates $X_1$, $X_2$, $X_4$, and $X_5$ are completely observed in the full cohort while covariates $X_3$ and $X_6$ are only observed in the case-cohort. We consider scenarios where the treatment variable *A* is completely observed in the full cohort or is only observed in the case-cohort.

Two full cohort sample sizes (3000 and 10000), two treatment effect sizes in RMST (zero and $17.76 \times 10^{-3}$), and two non-case to case ratios (1 and 2) are considered. For non-case to case ratio of 1 and 2, $40\%$ and $60\%$ of subjects in the full cohort are included in the case-cohort, respectively. The truncation time $\tau$ is set to 0.0614 and 0.1570 for the zero and nonzero treatment effect scenarios, respectively. We simulate 500 datasets for each simulation scenario. The true marginal treatment effect on RMST is determined by calculating the empirical difference between the potential RMST under treated and control from a large dataset of sample size. Both the true ATE and the true ATT are calculated as benchmarks for the methods examined in this simulation. The true ATE $\varDelta _{ATE} = \sum _{i=1}^{n} \frac{1}{n}(Z_i^1 - Z_i^0)$. The true ATT $\varDelta _{ATT} = \sum _{i=1}^{n} \frac{1}{n}I(A_i = 1)(Z_i^1 - Z_i^0)$.

### Estimation strategies

In this simulation study, we compare the following causal effect estimation methods.

#### Weighted Kaplan–Meier for case-cohort (CC)

This is the proposed method that uses the weighted Kaplan-Meier estimator $\hat{S}_{cc}(t)$ to estimate the marginal RMST under the stratified case-cohort design and propensity score stratification. In the simulation, the propensity score is estimated using the correct propensity score model with weighted logistic regression. Next, seven equally sized propensity strata are created. RMST is estimated based on $\hat{S}_{cc}(t)$ for the treated and control arm within each propensity score stratum. ATE and ATT are then calculated as the weighted average of the stratum-specific RMST difference. Since the CC method only uses the case-cohort sample, its performance is not affected by whether the treatment variable is observed only in the case-cohort or in the full cohort.

#### Multiple imputation (MI)

This method treats covariates (and treatment) not measured in the full cohort as missing data and imputes them before conducting a full cohort analysis (Van Buuren 2018). The missing data are imputed five times. In each of the five imputed full cohorts, the propensity score is estimated using the correct propensity score model with logistic regression. The propensity score is then stratified into seven strata, and the RMST is estimated based on the regular Kaplan-Meier estimator for the treated and control arm within each stratum of the full cohort. The ATE and ATT are then calculated for each of the five imputed full cohorts. Finally, Rubin’s rule is used to pool the estimated causal effects and their variances from the five datasets (Rubin 2004). The performance of this method is affected by whether the treatment variable is observed only in the case-cohort or in the full cohort since in the former scenario the treatment variable needs to be imputed.

The R package *mice* is used to perform multiple imputation. Continuous covariates are imputed using predictive mean matching (PMM). PMM is robust against misspecification of the imputation model and has been found to produce the least biased estimate among the available methods (Marshall et al. 2010). For binary variables, Bayesian logistic regression is used (Van Buuren 2018). We consider scenarios where the imputation model is specified incorrectly or correctly. A correctly specified imputation is defined as the imputation model that includes the log observed event time, event indicator, and all covariates in the correct functional form. The misspecified imputation model is defined as the model using the log observed event time, event indicator, and all covariates except $X_2$ and $X_5$.

As benchmarks, we also estimate ATE and ATT using the full cohort data with and without propensity score stratification.

### Performance evaluation

**Fig. 1:**
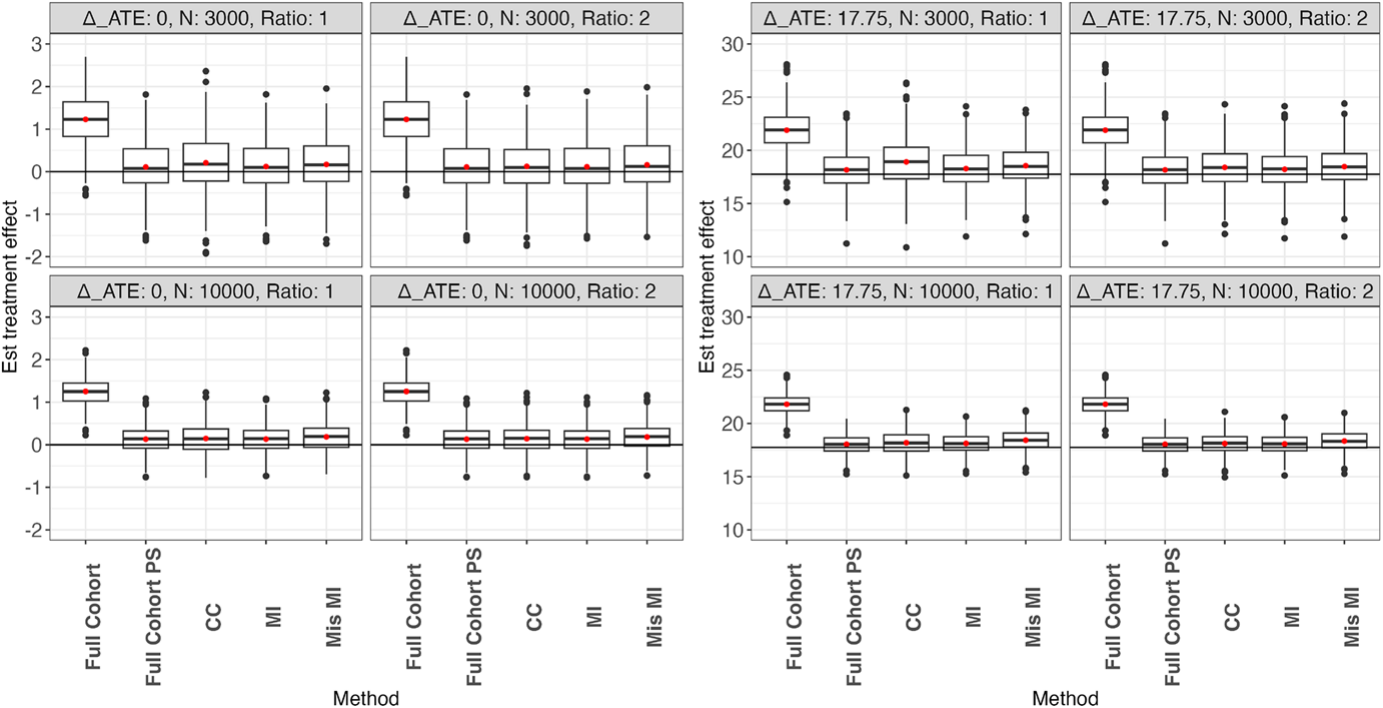
Boxplot of estimate of ATE where treatment is observed in the full cohort. The left and right panels are the scenarios with zero and nonzero treatment effect, respectively. Boxplot shows the median and IQR of the effect estimate, red dot shows the mean effect estimate, and horizontal line shows the true effect on RMST. Full Cohort estimates causal effect using full-cohort data without confounding adjustment. Full Cohort PS uses full-cohort data with confounding adjustment. CC uses case-cohort data with confounding adjustment. MI uses multiple-imputation to impute the case-cohort data with confounding adjustment. Mis-MI uses a misspecified multiple-imputation model to impute the case-cohort data with confounding adjustment

**Fig. 2:**
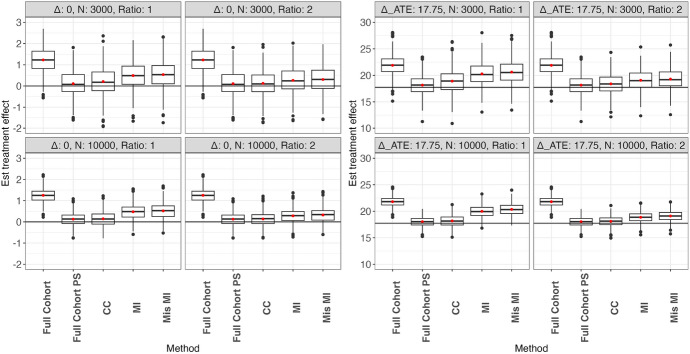
Boxplot of estimate of ATE where treatment is only observed in the case-cohort. The left and right panels are the scenarios with zero and nonzero treatment effect, respectively. Boxplot shows the median and IQR of the effect estimate, red dot shows the mean effect estimate, and horizontal line shows the true effect RMST. Full Cohort estimates causal effect using full-cohort data without confounding adjustment. Full Cohort PS uses full-cohort data with confounding adjustment. CC uses case-cohort data with confounding adjustment. MI uses multiple-imputation to impute the case-cohort data with confounding adjustment. Mis-MI uses a misspecified multiple-imputation model to impute the case-cohort data with confounding adjustment

The performance of causal effect estimation is measured by bias, percent bias, model-based standard error (SE), empirical standard error (ESE), and $95\%$ coverage probability (CP). The bias is defined as the estimated treatment effect minus the true effect. Percent bias is the bias of the estimate divided by the nonzero true treatment effect multiplied by 100. SE is the average of the estimated standard error based on the derived formula over all simulation replications. ESE is the standard deviation of the treatment effect estimates over all simulation replications. ESE is deemed as the true standard error. Finally, CP is the proportion of $95 \%$ confidence intervals in all replications that contain the true treatment effect (Figs. 1 and 2).

### Results

**Fig. 4:**
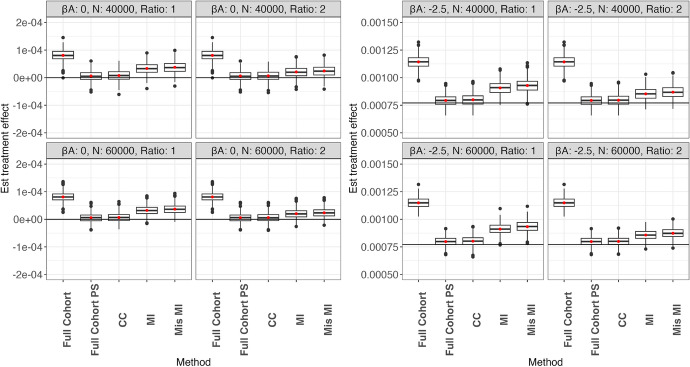
Boxplot of estimate of ATT where treatment is observed in the case-cohort and with 95$\%$ censoring rate. The left and right panels are the scenarios with zero and nonzero treatment effect, respectively. Boxplot shows the median and IQR of the effect estimate, red dot shows the mean effect estimate, and horizontal line shows the true effect RMST. Full Cohort estimates causal effect using full-cohort data without confounding adjustment. Full Cohort PS uses full-cohort data with confounding adjustment. CC uses case-cohort data with confounding adjustment. MI uses multiple-imputation to impute the case-cohort data with confounding adjustment. Mis-MI uses a mis-specified multiple-imputation model to impute the case-cohort data with confounding adjustment

The performance of estimating ATE for scenarios in which the treatment indicator is observed in the full cohort and only in the case-cohort is summarized in Figs. 4 and 2, respectively. Each figure contains scenarios with different treatment effects, sample sizes, and non-case to case ratios. Detailed numerical results on SE, ESE, and CP can be found in Table 1 and 2. Since the ATT estimation performance is similar to the ATE estimation performance, only the results of the ATE estimation are presented. The results of the ATT estimation can be found in Tables 5 and 6 in Appendix 1.4. Additional simulation results with 95% censoring rate can be found in Appendix 1.5.

**Table 1:** Performance of estimation methods for the marginal RMST treatment effect for the population (ATE) under the scenario where treatment is observed for the full cohort. Est. TE, ESE, and SE are multiplied by 1,000 for ease of presentation. Full Cohort estimates causal effect using full-cohort data without confounding adjustment. Full Cohort PS uses full-cohort data with confounding adjustment. CC uses case-cohort data with confounding adjustment. MI uses multiple-imputation to impute the case-cohort data with confounding adjustment. Mis-MI uses a misspecified multiple-imputation model to impute the case-cohort data with confounding adjustment

N	Ratio	Method	Est.	Abs. bias	ESE	SE	CP
			$\beta _A = 0$, $\varDelta _{ATE}=0$				
3000	1	Full Cohort	1.24	1.24	0.61	0.61	0.49
3000	1	Full Cohort PS	0.11	0.11	0.60	0.65	0.97
3000	1	CC	0.21	0.21	0.71	0.66	0.92
3000	1	MI	0.12	0.12	0.60	0.66	0.98
3000	1	Mis MI	0.17	0.17	0.61	0.67	0.97
3000	2	Full Cohort	1.24	1.24	0.61	0.61	0.49
3000	2	Full Cohort PS	0.11	0.11	0.60	0.65	0.97
3000	2	CC	0.12	0.12	0.62	0.61	0.95
3000	2	MI	0.11	0.11	0.60	0.65	0.97
3000	2	Mis MI	0.16	0.16	0.61	0.66	0.96
10000	1	Full Cohort	1.25	1.25	0.33	0.33	0.02
10000	1	Full Cohort PS	0.13	0.13	0.32	0.34	0.95
10000	1	CC	0.15	0.15	0.36	0.35	0.93
10000	1	MI	0.13	0.13	0.32	0.34	0.95
10000	1	Mis MI	0.18	0.18	0.32	0.35	0.94
10000	2	Full Cohort	1.25	1.25	0.33	0.33	0.02
10000	2	Full Cohort PS	0.13	0.13	0.32	0.34	0.95
10000	2	CC	0.14	0.14	0.33	0.33	0.94
10000	2	MI	0.13	0.13	0.32	0.34	0.95
10000	2	Mis MI	0.18	0.18	0.32	0.34	0.94

N	Ratio	Method	Est.	% bias	ESE	SE	CP
			$\beta _A=-2.5$, $\varDelta _{ATE}=17.75$				
3000	1	Full Cohort	21.91	23.38	2.00	1.93	0.42
3000	1	Full Cohort PS	18.16	2.29	1.91	1.96	0.94
3000	1	CC	18.92	6.56	2.29	2.13	0.93
3000	1	MI	18.29	3.00	1.90	2.02	0.96
3000	1	Mis MI	18.56	4.53	1.93	2.06	0.96
3000	2	Full Cohort	21.91	23.38	2.00	1.93	0.42
3000	2	Full Cohort PS	18.16	2.29	1.91	1.96	0.94
3000	2	CC	18.40	3.65	2.00	1.91	0.93
3000	2	MI	18.22	2.60	1.91	1.98	0.94
3000	2	Mis MI	18.47	4.06	1.95	2.02	0.95
10000	1	Full Cohort	21.81	22.82	0.95	1.05	0.01
10000	1	Full Cohort PS	18.04	1.59	0.90	1.01	0.97
10000	1	CC	18.18	2.42	1.07	1.11	0.95
10000	1	MI	18.12	2.05	0.90	1.03	0.96
10000	1	Mis MI	18.42	3.74	0.94	1.06	0.94
10000	2	Full Cohort	21.81	22.82	0.95	1.05	0.01
10000	2	Full Cohort PS	18.04	1.59	0.90	1.01	0.97
10000	2	CC	18.11	1.98	0.97	1.03	0.94
10000	2	MI	18.07	1.77	0.90	1.02	0.97
10000	2	Mis MI	18.34	3.29	0.93	1.04	0.95

**Table 2:** Performance of estimation methods for the marginal RMST treatment effect for the population (ATE) under the scenario where treatment is only observed for the case-cohort. Est. TE, bias, ESE, and SE are multiplied by 1,000 for ease of presentation. Full Cohort estimates causal effect using full-cohort data without confounding adjustment. Full Cohort PS uses full-cohort data with confounding adjustment. CC uses case-cohort data with confounding adjustment. MI uses multiple-imputation to impute the case-cohort data with confounding adjustment. Mis-MI uses a misspecified multiple-imputation model to impute the case-cohort data with confounding adjustment

N	R	Method	Est.	Abs. bias	ESE	SE	CP
			$\beta _A = 0$, $\varDelta _{ATE}=0$				
3000	1	Full Cohort	1.24	1.24	0.61	0.61	0.49
3000	1	Full Cohort PS	0.11	0.11	0.60	0.65	0.97
3000	1	CC	0.21	0.21	0.67	0.66	0.93
3000	1	MI	0.49	0.49	0.66	0.72	0.93
3000	1	Mis MI	0.53	0.53	0.67	0.74	0.92
3000	2	Full Cohort	1.24	1.24	0.61	0.61	0.49
3000	2	Full Cohort PS	0.11	0.11	0.60	0.65	0.97
3000	2	CC	0.15	0.15	0.64	0.61	0.93
3000	2	MI	0.27	0.27	0.62	0.68	0.97
3000	2	Mis MI	0.31	0.31	0.63	0.69	0.96
10000	1	Full Cohort	1.25	1.25	0.33	0.33	0.02
10000	1	Full Cohort PS	0.13	0.13	0.32	0.34	0.95
10000	1	CC	0.13	0.13	0.34	0.35	0.95
10000	1	MI	0.48	0.48	0.35	0.37	0.79
10000	1	Mis MI	0.52	0.52	0.36	0.38	0.76
10000	2	Full Cohort	1.25	1.25	0.33	0.33	0.02
10000	2	Full Cohort PS	0.13	0.13	0.32	0.34	0.95
10000	2	CC	0.12	0.12	0.33	0.34	0.95
10000	2	MI	0.29	0.29	0.33	0.35	0.90
10000	2	Mis MI	0.33	0.33	0.34	0.36	0.87

N	R	Method	Est.	% bias	ESE	SE	CP
			$$\beta _A=-2.5,$$$$\varDelta _{ATE}=17.75$$				
3000	1	Full Cohort	21.91	23.38	2.00	1.93	0.42
3000	1	Full Cohort PS	18.16	2.29	1.91	1.96	0.94
3000	1	CC	18.84	6.06	2.20	2.13	0.93
3000	1	MI	20.28	14.25	2.15	2.36	0.86
3000	1	Mis MI	20.62	16.13	2.20	2.41	0.81
3000	2	Full Cohort	21.91	23.38	2.00	1.93	0.42
3000	2	Full Cohort PS	18.16	2.29	1.91	1.96	0.94
3000	2	CC	18.36	3.34	1.97	1.91	0.94
3000	2	MI	19.08	7.46	1.97	2.13	0.95
3000	2	Mis MI	19.30	8.72	2.00	2.17	0.93
10000	1	Full Cohort	21.81	22.82	0.95	1.05	0.01
10000	1	Full Cohort PS	18.04	1.59	0.90	1.01	0.97
10000	1	CC	18.19	2.39	1.08	1.11	0.95
10000	1	MI	19.99	12.58	1.07	1.21	0.57
10000	1	Mis MI	20.35	14.64	1.10	1.24	0.43
10000	2	Full Cohort	21.81	22.82	0.95	1.05	0.01
10000	2	Full Cohort PS	18.04	1.59	0.90	1.01	0.97
10000	2	CC	18.07	1.75	1.00	1.03	0.95
10000	2	MI	18.87	6.28	0.96	1.10	0.88
10000	2	Mis MI	19.12	7.69	0.98	1.12	0.82

Using the full cohort without propensity score stratification (Full Cohort), the estimated RMST difference is highly biased in all scenarios due to confounding. With propensity score stratification on the full cohort (Full Cohort PS), a majority of the bias is removed, indicating that propensity score stratification is effective in adjusting for confounding bias. However, the remaining bias in Full Cohort PS indicates the existence of residual confounding not accounted for by propensity score stratification. The proposed method based on the weighted Kaplan-Meier estimator for the stratified case-cohort sample (CC) performs similarly to Full Cohort PS in terms of bias, but with a larger ESE in all scenarios. The increased ESE in CC method is expected as it uses less data than Full Cohort PS. In both CC and Full Cohort PS, the SE is reasonably close to ESE and the CP is reasonably close to the nominal level considering the slightly biased point estimates due to residual confounding. Increasing the non-case to case ratio reduces the bias and improves the SE and CP of the CC method, especially for smaller sample sizes.

In scenarios where treatment is observed for all subjects, the correctly specified multiple imputation method (MI) has slightly smaller bias, similar CP, and smaller ESE than the CC method. The smaller ESE is due to the fact that MI makes better use of fully observed covariates than the CC method. When the multiple imputation model is misspecified (Mis MI) by omitting covariates $X_2$ and $X_5$, the bias increases and becomes greater than that in the CC method.

In the scenario where treatment is only observed for the case-cohort subjects, both MI and Mis MI have a much poorer performance than the CC method in all evaluation indices. This is likely due to the misclassification of imputed treatment during the imputation stage, which leads to biased propensity score estimates and in turn biased PS stratification. The performance of both MI and Mis MI substantially improves with increased non-case to case ratio since treatment needs to be imputed in fewer subjects, but their performance is still much worse than that of the CC method.

In summary, the proposed CC method gives the best overall causal effect estimation across all scenarios and therefore seems to be the most robust one to use when estimating causal effects on RMST based on case-cohort data. As the total sample size and non-case to case ratio increase, the precision of the estimated causal effects improves. Multiple imputation-based methods suffer from imputation model misspecification and biased propensity score estimation due to misclassification of imputed treatment.

To evaluate the impact of the number of PS strata on the performance of the proposed method, we performed additional simulations using 5, 7, and 10 PS strata. The results are included in Appendix 1.6. It seems that seven PS strata are adequate to remove most of the confounding bias. Any additional strata do not significantly reduce bias further.

Additional simulation results under an unstratified case-cohort design can be found in the Appendix 1.7. The performance of the proposed method is very similar to that under a stratified case-cohort design, confirming the applicability of the proposed method to both the unstratified and stratified case-cohort design.

### Real data analysis (ARIC study)

The ARIC study monitored 15,792 individuals from four U.S. communities for cardiovascular events and mortality over several decades (Investigators 1989; Ballantyne et al. 2004). After excluding subjects with missing data, the full cohort includes 12,197 individuals. Those who survived and remained disease-free by the end of 1998, or who were lost to follow-up, were considered censored. A random subcohort of 978 individuals was selected through stratified random sampling based on sex, race (black vs. white) and age at baseline ($\le 55$ years vs. $>55$ years). The study documented 638 CHD cases or CHD-related deaths, resulting in a censoring rate of $94.8\%$. After including all CHD cases, the case-cohort consisted of 1,567 subjects. The purpose of this analysis is to determine the average treatment effect (ATE) and average treatment effect on the treated (ATT) for individuals with hs-CRP levels $>3$ compared to those with levels $\le$ 3 with respect to RMST of CHD. To calculate the RMST for each hs-CRP group, a truncation time $\tau$ of 8 years (2,920 days) was chosen, given the duration of follow-up. Table 3 presents summary statistics of the baseline variables, highlighting the imbalance of the covariate distribution between the high and low hs-CRP groups, which necessitates adjustment for confounding. RMST was estimated using the proposed weighted method and multiple imputation on the case-cohort data. Given the study design, multiple imputation was only necessary for the CRP level (high / low), as the CRP level was the only unobserved covariate in the full cohort. Additionally, the ARIC study was analyzed using the Cox proportional hazards model. All methods incorporated propensity score stratification with seven strata.

For weighted analysis, each individual in the subcohort was weighted by their case-cohort sampling probability. When applying MI, we imputed the CRP level using the Bayesian logistic regression (logreg) method found in the *mice* library in R (van Buuren and Groothuis-Oudshoorn 2011). Imputation for the CRP level was performed using information from all baseline characteristics, the log of observation time, and the event indicator. The propensity score was calculated using a weighted logistic regression on the case-cohort sample with all baseline covariates. The balance between low and high CRP groups within each propensity score stratum was examined. Ideally, the standardized mean difference for each covariate within each propensity score stratum should be less than 0.1. We observed an acceptable balance for most covariates within each propensity score stratum. However, we observed some imbalance with a standardized mean difference exceeding 0.1 between the treatment and control arms in certain variable-strata combinations for both the case-cohort and the imputed data. Achieving perfect balance within each propensity score stratum is challenging due to the coarse nature of propensity score stratification.

Based on the results presented in Table 4, the estimated ATE is much larger in absolute value in CC than in MI. The SE is larger in CC, which is consistent with the observation in the simulation study. However, as we also observed in the simulation study, MI estimates are likely to be biased. The $95\%$ CI of the weighted method does not cover zero, indicating a significant difference in RMST between the lower and high CRP groups. The MI method did not find a significant difference in RMST. The estimated ATT and its SE are higher than the ATE for both methods. For ATT, neither method found a significant causal effect of high CRP on CHD RMST in those with high CRP. Finally, the stratified Cox proportional hazards model did not find a significant difference in terms of the hazard of CHD between the two CRP groups.

It is worth mentioning that the theoretical properties of the proposed method were established under the assumption of Bernoulli sampling, while the ARIC study uses simple random sampling. However, given that the random subcohort only represents about eight percent of the full cohort, the results from Bernoulli sampling and simple random sampling is expected to be very similar.

**Table 3:** Summary statistics of baseline variables by hs-CRP protein level between 0 and 3 mg/L and greater than 3 mg/L

Variable	hs-CRP level $\le$ 3 Mean (SD) or n (%)	hs-CRP level >3 Mean (SD) or n (%)
n	947	620
Age (years)	58.16 (5.59)	58.73 (5.42)
Current smoker (%)	203 (21.4)	197 (31.8)
Diabetes (%)	156 (16.5)	202 (32.6)
BMI	27.00 (4.20)	30.42 (6.23)
LDL (mg/dL)	138.05 (37.82)	141.67 (39.06)
HDL (mg/dL)	48.41 (16.00)	44.59 (14.73)
Systolic blood pressure (mmHg)	124.22 (19.25)	130.40 (21.06)
Hypertension (%)	0.37 (0.48)	0.57 (0.50)
Triglyceride (mmol/L)	128.40 (65.06)	145.45 (67.88)
Diastolic blood pressure (mmHg)	73.46 (11.07)	74.30 (10.87)

**Table 4:** Estimated effect of CRP level on the 8-year RMST of coronary heart disease (CHD) in days

Method	ATE (SE)	ATE 95% CI	ATT (SE)	ATT 95% CI
CC	$-$19.86 (9.37)	($-$38.24, $-$1.49)	$-$20.58 (11.14)	($-$42.42, 1.26)
MI	$-$11.51 (8.17)	($-$27.52, 4.51)	$-$13.83 (10.5)	($-$34.4, 6.74)
HR	1.24 (0.18)	(0.94, 1.64)	1.15 (0.15)	(0.89, 1.49)

### Discussion

In this paper, we introduce propensity score stratified estimation of the marginal causal effect measured by the RMST difference under a stratified case-cohort design. RMST difference has the advantage of not relying on the proportional hazards assumption and allows for the estimation of the marginal treatment effect. We propose RMST difference as an alternative causal effect measure to the commonly used HR in epidemiological studies.

RMST and HR, while both applicable to survival outcomes, measure different aspects of treatment effects. Specifically, RMST measures the effects on the additive scale when HR measures the effects on the multiplicative scale. In rare disease studies, one may observe a large fold-change in the hazards (i.e., large HR) but a relatively small RMST difference. This suggests that while the treated group may have a much higher risk of disease than the control group in terms of fold change, it may not correspond to a large change in the expected survival time for the population studied. Therefore, the RMST difference provides complementary insights that are not discernible from HR alone. In addition, the RMST difference offers a more intuitive interpretation, making it easier for clinicians and patients to grasp, as it represents the difference in life expectancy over a specific time horizon.

It is important to note that the definition of cases in this paper differs slightly from that of the stratified case-cohort design proposed by Borgan et al. (2000). The new definition of cases is unique to using RMST as an effect measure, as it includes all subjects who survive up to $\tau$ as cases, making better use of the information when estimating RMST. However, this new definition depends on the choice of $\tau$. Thus, it may not be applicable when analyzing an existing case-cohort dataset where the case-cohort sample has already been drawn according to the conventional definition of case, as is the case in the ARIC study. Moreover, when $\tau$ is small, there may be a large number of cases based on the new definition. In this case, a generalized case-cohort design could be considered, where only a random subset of unselected cases is included in the random subcohort to form the generalized case-cohort sample (Cai and Zeng 2007).

The independence between survival and censoring times in Assumption 3 is only conditioned on treatment *A* but not on covariates *X*. This is slightly stronger than the common assumption of independence conditional on *A* and *X* in the literature. This is because our proposed method stratifies subjects by propensity score rather than by covariates directly. Therefore, even if subjects have the same propensity scores, their covariate values can still be different. This difference may result in residual dependence between survival and censoring times within PS strata and thereby introduce bias in the RMST estimator. However, even if independence can only be achieved by conditioning on both *A* and *X* (thus violating Assumption 3), as the number of PS strata increases with the sample size, we expect the bias in the RMST estimator due to residual dependent censoring to become very small.

An important practical question is how to choose the truncation time $\tau$. Ideally, $\tau$ should be chosen by the investigator before the analysis based on clinical justification. Tian et al. (2020) proposed a data-driven approach to select $\tau$, which could be adapted to a stratified case-cohort design.

In this paper, we proposed to estimate propensity scores using a weighted logistic regression to account for the case-cohort sampling design. Other machine methods such as bagging or boosting, random forests, and neural networks have been used to estimate propensity scores in the literature (Lee et al. 2010; Setoguchi et al. 2008; McCaffrey et al. 2004)5. It is an interesting future research topic to extend these methods to a stratified case-cohort design so that they can be used to estimate propensity scores in the settings considered in this paper.

One limitation of our approach is that propensity score stratification can only address observed confounding bias, leaving hidden bias from unobserved confounders unaccounted for. To mitigate this issue, sensitivity analysis methods, such as discussed in Rosenbaum et al. (2010), could be extended to a stratified case-cohort design. This presents another topic for future research.

## Figures and Tables

**Fig. 3:**
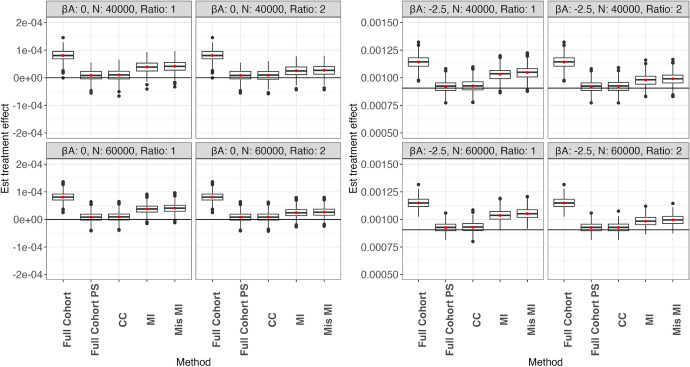
Boxplot of estimate of ATE where treatment is observed in the case-cohort and with 95$\%$ censoring rate. The left and right panels are the scenarios with zero and nonzero treatment effect, respectively. Boxplot shows the median and IQR of the effect estimate, red dot shows the mean effect estimate, and horizontal line shows the true effect RMST. Full Cohort estimates causal effect using full-cohort data without confounding adjustment. Full Cohort PS uses full-cohort data with confounding adjustment. CC uses case-cohort data with confounding adjustment. MI uses multiple-imputation to impute the case-cohort data with confounding adjustment. Mis-MI uses a mis-specified multiple-imputation model to impute the case-cohort data with confounding adjustment

**Fig. 5:**
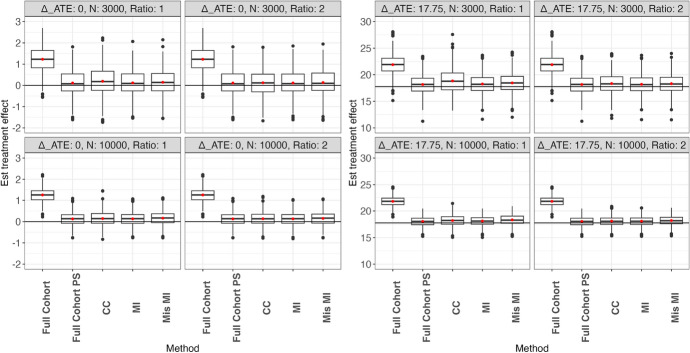
Boxplot of estimate of ATE where treatment is observed in the full cohort. Data is generated from a case-cohort design with no stratification. The left and right panels are the scenarios with zero and nonzero treatment effect, respectively. Boxplot shows the median and IQR of the effect estimate, red dot shows the mean effect estimate, and horizontal line shows the true effect on RMST. Full Cohort estimates causal effect using full-cohort data without confounding adjustment. Full Cohort PS uses full-cohort data with confounding adjustment. CC uses case-cohort data with confounding adjustment. MI uses multiple-imputation to impute the case-cohort data with confounding adjustment. Mis-MI uses a misspecified multiple-imputation model to impute the case-cohort data with confounding adjustment

**Fig. 6:**
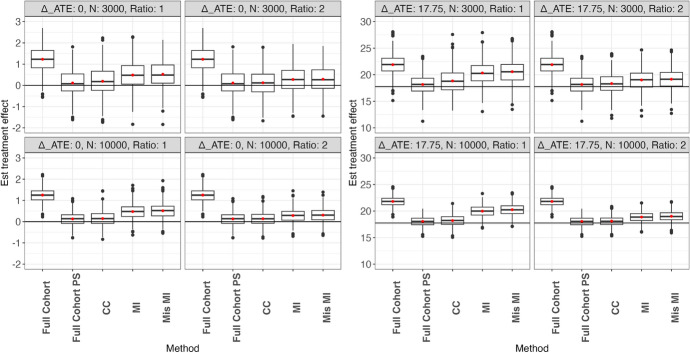
Boxplot of estimate of ATE where treatment is only observed in the case cohort. Data is generated from a case-cohort design with no stratification. The left and right panels are the scenarios with zero and nonzero treatment effect, respectively. Boxplot shows the median and IQR of the effect estimate, red dot shows the mean effect estimate, and horizontal line shows the true effect on RMST. Full Cohort estimates causal effect using full-cohort data without confounding adjustment. Full Cohort PS uses full-cohort data with confounding adjustment. CC uses case-cohort data with confounding adjustment. MI uses multiple-imputation to impute the case-cohort data with confounding adjustment. Mis-MI uses a misspecified multiple-imputation model to impute the case-cohort data with confounding adjustment

## Appendix 1

### Appendix 1.1: Proof of Proposition 1

#### Proof

Let there be *N* subjects in the full cohort. Assume that we observe a binary treatment variable $\{A_i: i=1,\dots ,N\}$ following a Bernoulli distribution with parameter $p_i=e^{\varvec{x}_i'\beta }(1+e^{\varvec{x}_i'\beta })^{-1}$ under a logistic regression model, where $\varvec{x}_i$ is a p-dimensional covariate vector for observation *i*. $p_i$ is the true propensity score for subject *i*. Under a case-cohort design, $y_i$ and $\varvec{x}_i$ are only fully observed in the case-cohort sample. Therefore, in logistic regression, each subject should be weighted by the case-cohort sampling weights $\{\rho _i = \delta _i + (1-\delta _i)\frac{\xi _i}{\alpha _i}: i=1,...,N\}$ where $\delta _i$ is the event indicator, $\xi _i$ is the random subcohort membership indicator and $\alpha _i$ is the random subcohort sampling probability. The log-likelihood of the weighted logistic regression is $l^w(\beta \mid A) = \sum _{i=1}^N A_i \rho _i \varvec{x}_i' \beta - \sum _{i=1}^N \rho _i \log (1+e^{\varvec{x}_i'\beta })$. The score function is defined as

$$\begin{aligned} U_j^w(\beta )&= \frac{l^w(\beta \mid A)}{d\beta _j} \\&= \sum _{i=1}^N A_i \rho _i x_{ij} - \sum _{i=1}^N \frac{\rho _i x_{ij} e^{\varvec{x}_i' \beta }}{1 + e^{\varvec{x}_i' \beta }} \\&= \sum _{i=1}^N (A_i \rho _i x_{ij} - \rho _i p_i x_{ij}) \\ \textrm{E}\Big ( U_j^w(\beta ) \Big )&= \sum _{i=1}^N \textrm{E}(A_i \rho _i x_{ij} - p_i x_{ij}) \\&= \sum _{i=1}^n x_{ij} \left( \textrm{E}(A_i \rho _i) - p_i \textrm{E}(\rho _i) \right) . \end{aligned}$$

Since $$\textrm{E}(\xi _i)=\alpha _i$$ and $$\textrm{Var}(\xi _i) = (1-\alpha _i)\alpha _i$$,

$$\begin{aligned} \textrm{E}(A_i \rho _i)&= \textrm{E}\big (\textrm{E}(A_i \rho _i \mid A_i)\big ) \\&= \textrm{E}\big (\textrm{E}(A_i(\delta _i + (1-\delta _i)\frac{\xi _i}{\alpha _i}) \mid A_i)\big ) \\&= \textrm{E}\big (A_i \textrm{E}(\delta _i + (1-\delta _i) \frac{\xi _i}{\alpha _i} \mid A_i)\big ) \\&= \textrm{E}\big (A_i (\delta _i + (1-\delta _i)\frac{\alpha _i}{\alpha _i})\big ) \\&= \textrm{E}(A_i) \\&= p_i. \end{aligned}$$

By the same argument, $\textrm{E}(\rho _i) = 1$.

Let $\hat{\beta }$ be the estimated regression coefficient obtained maximizing the log-likelihood and $\beta _0$ be the true regression coefficient. Since $\textrm{E}\big ( U^w_j(\beta _0)\big ) = \sum _{i=1}^N x_{ij}(p_i - p_i) = 0$ for $j=1,\dots ,p$, $\hat{\beta } \rightarrow \beta _0$ in probability. Let $\hat{p}_i = e^{\varvec{x}_i'\hat{\beta }}(1+e^{\varvec{x}_i'\hat{\beta }})^{-1}$ be the estimated propensity score for subject *i*. By Slutsky’s Theorem, $\hat{p}_i \rightarrow p_i$ in probability for all *i*. Therefore, weighted logistic regression will produce consistent estimates of $\beta$ using the individual case-cohort weight, which leads to consistent estimates of propensity scores.$\square$

### Appendix 1.2: Proof of Theorem 1

#### Proof

Assume that there are *B* case-cohort strata. Let there be *N* subjects in the full cohort. Assume that the sampling probability of the random subcohort in the case-cohort stratum *b* is $\alpha ^b$, then the case-cohort weight for subject $i=1,...,N$ is $\rho _i = \delta _i + (1-\delta _i)\frac{\xi _i}{\alpha _i}$ where $\delta _i$ is the event indicator, $\xi _i$ is the random subcohort membership indicator, and $\alpha _i = \alpha ^b$ if subject *i* belongs to the case-cohort stratum *b*.

We will estimate *S*(*t*) using the modified Kaplan-Meier estimator under the case-cohort design, $\hat{S}_{cc}(t) = \exp \{-\int _0^t d\hat{\varLambda }_{cc}(u) \} = \exp (-\hat{\varLambda }_{cc}(t))$ where $d\hat{\varLambda }_{cc}(u) = \frac{\sum _{i=1}^N dN_i(u)}{\sum _{j=1}^N Y_j(u)\rho _j}$. To establish the asymptotic properties of $\hat{S}_{cc}(t)$, we first establish the asymptotic property of $\hat{\varLambda }_{cc}(t)$.

$$\begin{aligned} \sqrt{N}(\hat{\varLambda }_{cc}(t) - \varLambda _0(t)) =&\sqrt{N}\big (\int _0^t \frac{\sum _{i=1}^N dN_i(u)}{\sum _{j=1}^N Y_j(u) \rho _j }- \varLambda _0(t)\big )\\ =&\int _0^t \frac{N}{\sum _{j=1}^N Y_j(u) \rho _j} \frac{1}{\sqrt{N}} d\sum _{i=1}^N M_i(u) \\&\quad + \int _0^t \frac{N}{\sum _{j=1}^N Y_j(u) \rho _j} \frac{1}{\sqrt{N}} \sum _{i=1}^n Y_i(u) d\varLambda _0(u) \\&\quad - \sqrt{N}\int _0^t d\varLambda _0(u) \\ =&\int _0^t \frac{N}{\sum _{j=1}^N Y_j(u) \rho _j} \frac{1}{\sqrt{N}} d\sum _{i=1}^N M_i(u) \\&\quad + \sqrt{N} \int _0^t \frac{\sum _{i=1}^N Y_i(u) - \sum _{j=1}^N Y_j(u) \rho _j}{\sum _{j=1}^N Y_j(u) \rho _j} d\varLambda _0(u) \end{aligned}$$

where $dM_i(u) = dN_i(u) - Y_i(u)d\varLambda _i(u)$ is a martingale by Doob Meyer decomposition.

Let $\mathcal {F}(t)$ be the sigma algebra generated by $N_j(u)$, $Y_j(u)$ and $\delta _j$ for $0 \le u \le t$ and $j=1,...,n$, then $\textrm{E}\big [\xi \mid \mathcal {F}(t)\big ]=\alpha$ and $\textrm{Var}\big [\xi \mid \mathcal {F}(t)\big ] = (1-\alpha )\cdot \alpha$. Under Bernoulli sampling, $\xi _i$’s are independent of each other. By weak law of large numbers, $\frac{1}{N}\sum _{j=1}^N Y_j(u)\rho _j \xrightarrow {p} \pi (u)$ where $\pi (u)=\textrm{E}[Y(u)\rho ]= \textrm{E}[Y(u)\textrm{E}(\rho \mid \mathcal {F}(t))] = \textrm{E}(Y(u))$ since $\textrm{E}(\rho \mid \mathcal {F}(t)) = 1$. It follows that

$$\begin{aligned} \sqrt{N} \big (\hat{\varLambda }_{cc}(t) - \varLambda _0(t) \big ) =&\frac{1}{\sqrt{N}} \Big \{\int _0^t \sum _{i=1}^N \big [ \frac{N}{\sum _{j=1}^N Y_j(u)\rho _j} - \pi ^{-1}(u) + \pi ^{-1}(u)\big ]dM_i(u) \\&\quad + \int _0^t \sum _{j=1}^N Y_j(u)(1-\delta _j)(1-\frac{\xi _j}{\alpha _j}) \\&\quad \quad \times \big [ \frac{N}{ \sum _{j=1}^N Y_j(u)\rho _j} - \pi ^{-1}(u) + \pi ^{-1}(u)\big ]d\varLambda _0(u) \Big \} \end{aligned}$$

has the same asymptotic distribution as

$$\begin{aligned} \frac{1}{\sqrt{N}} \int _0^t \sum _{i=1}^N \pi ^{-1}(u) dM_i(u)&+ \frac{1}{\sqrt{N}} \int _0^t \sum _{j=1}^N Y_j(u)(1-\delta _j)(1-\frac{\xi _j}{\alpha _j}) \pi ^{-1}(u) d\varLambda _0(u) \\&= I_1 + I_2. \end{aligned}$$

Let

$$\begin{aligned} I_1 = \frac{1}{\sqrt{N}} \int _0^t \sum _{i=1}^N \pi ^{-1}(u) dM_i(u) \end{aligned}$$

and

$$\begin{aligned} I_2 = \sum _{b=1}^B I_{2b} = \sum _{b=1}^B \sqrt{p_b}\frac{1}{\sqrt{N_b}}\int _0^t \sum _{j=1}^{N_b} Y_j(u)(1-\delta _j)(1-\frac{\xi _j}{\alpha _j}) \pi ^{-1}(u) d\varLambda _0(u) \end{aligned}$$

where $p_b= \frac{N_b}{N}$ and $N_b$ is the number of subjects in the stratified subcohort $b$.

Since $M_i(u)$ is a martingale and $\pi ^{-1}(u)$ is a bounded deterministic process, by the Martingale Central Limit Theorem, $I_1$ converges weakly to a Wiener process with covariance process

$$\begin{aligned} \sigma _1^2(t) = \int _0^t \pi ^{-1}(u)d\varLambda _0(u). \end{aligned}$$

Consider the term $I_{2b}$ for $b=1,...B$,

$$\begin{aligned} I_{2b}&= \sqrt{p_b}\frac{1}{\sqrt{N_b}}\sum _{j=1}^{N_b}\int _0^tY_j(u)(1-\delta _j)(1-\frac{\xi _j}{\alpha _b})\pi ^{-1}(u) d\varLambda _0(u). \end{aligned}$$

Since $\int _0^t Y_j(u)(1-\delta _j)(1-\frac{\xi _j}{\alpha _b})\pi ^{-1}(u)d\varLambda _0(u)$ are iid random processes with bounded variation, applying the Central Limit Theorem at each time *t*, we know that $\frac{1}{\sqrt{N_b}} \sum _{j=1}^{N_b} \int _0^t Y_j(u)(1-\delta _j)(1-\frac{\xi _j}{\alpha _b})\pi ^{-1}(u)d\varLambda _0(u)$ converges pointwise in distribution to a normal distribution with mean $\mu (t) = \textrm{E}\big [\int _0^t Y(u)(1-\delta )(1-\frac{\xi _j}{\alpha _b})\pi (u)^{-1}d\varLambda _0(u)\big ]$. The tightness of the process can be proved by following Example 2.11.16 from Wellner (2013). Thus, $I_{2b}$ converges weakly to a Wiener process with mean $\sqrt{p_b}\mu (t)$ and covariance process $\sigma _2^2(t)=\textrm{Var}\Big [\sqrt{p_b}\int _0^tY_j(u)(1-\delta _j)(1-\frac{\xi _j}{\alpha _b})\pi ^{-1}(u) d\varLambda _0(u) \Big ] = \textrm{Var}\Big [\sqrt{p_b}(1-\frac{\xi _j}{\alpha _b})\int _0^tY_j(u)(1-\delta _j)\pi ^{-1}(u) d\varLambda _0(u) \Big ]$.

Thus,

$$\begin{aligned} \sigma _2^2(t)&= \textrm{E}\big \{ \textrm{Var}\big [ \sqrt{p_b} (1-\frac{\xi _j}{\alpha _b})\int _0^t Y_j(u)(1-\delta _j)\pi (u)^{-1} d\varLambda _0(u) \mid \mathcal {F}(t)\big ] \big \} \\&\qquad + \textrm{Var}\big \{ E\big [\sqrt{p_b} (1-\frac{\xi _j}{\alpha _b})\int _0^t Y_j(u)(1-\delta _j)\pi (u)^{-1} d\varLambda _0(u) \mid \mathcal {F}(t)\big ] \big \} \\&= p_b \frac{1-\alpha _b}{\alpha _b} \cdot \textrm{E}\big [ \int _0^t Y_j(u) (1-\delta _j)\pi (u)^{-1}d\varLambda _0(u)\big ]^2 + 0. \end{aligned}$$

Since $$\textrm{E}(I_1) = 0$$ and $$\textrm{E}(I_2) = 0$$,

$$\begin{aligned} \textrm{cov}(I_1,I_2)&= \textrm{E}(I_1 I_2) \\&= \textrm{E}\big [I_1 \textrm{E}(I_2\mid \mathcal {F}(t)) \big ] \\&= \textrm{E}\big [I_1 \int _0^t \frac{1}{\sqrt{N}} \sum _{j=1}^N Y_j(u)(1-\delta _j)(1-\frac{\textrm{E}(\xi _j \mid \mathcal {F}(t))}{\alpha _j}\pi ^{-1}(u) dA_0(u) \big ] \\&= 0. \end{aligned}$$

Thus, $$I_1$$ is independent of $$I_2$$. Therefore, $$\sqrt{N}\big (\hat{\varLambda }_{cc}(t) - \varLambda _0(t)\big )$$ converges to a Wiener process with covariance process $$\sigma _{1}^{2} (t) + \sigma _{2}^{2} (t) = \int_{0}^{t} {\pi ^{{ - 1}} } (u)d\Lambda _{0} (u) + (\sum\limits_{{b = 1}}^{B} {p_{b} } \frac{{1 - \alpha _{b} }}{{\alpha _{b} }})$$$$E[\int_{0}^{t} {Y_{j} } (u)(1 - \delta _{j} )\pi ^{{ - 1}} (u)d\Lambda _{0} (u)]^{2} \nu$$. By Theorem II.8.1 of Andersen et al. (1993) $$\sqrt{N}\big (\hat{S}_{cc}(t) - S_0(t)\big )$$ is asymptotically equivalent to $$-S_0(t)\sqrt{N}\big (\hat{\varLambda }_{cc}(t) - \varLambda _0(t)\big )$$. Therefore, $$\sqrt{N}\big (\hat{S}_{cc}(t) - S_0(t)\big )$$ converges to a Wiener process with covariance process $$S_0^2(t)\Big \{\int _0^t \pi ^{-1}(u)d\varLambda _0(u) + (\sum _{b=1}^B p_b \frac{1-\alpha _b}{\alpha _b})\textrm{E}\big [\int _0^t Y_j(u)(1-\delta _j)\pi ^{-1}(u)d\varLambda _0(u) \big ]^2\Big \}$$.

Let $\mu _0(\tau ) = \int _0^\tau S_0(u) du$ and $\hat{\mu }_{cc}(\tau ) = \int _0^\tau \hat{S}_{cc}(u) du$, then

$$\begin{aligned} \sqrt{N}\big (\hat{\mu }_{cc}(\tau ) - \mu _0(\tau ) \big )&= \int _0^\tau \sqrt{N}\big (\hat{S}_{cc}(u) - S_0(u) \big ) du \end{aligned}$$

is asymptotically equivalent to

$$\begin{aligned} -\int _0^\tau S_0(u)\sqrt{N}(\hat{\varLambda }_{cc}(u) - \varLambda _0(u))du&= \int _0^\tau \sqrt{N}(\hat{\varLambda }_{cc}(u)-\varLambda _0(u)) d\overline{\mu }(\tau ,u) \\&= -\int _0^\tau \overline{\mu }(\tau ,u)\sqrt{N}(d\hat{\varLambda }_{cc}(u) - d\varLambda _0(u)). \end{aligned}$$

Since $\sqrt{N}\big (\hat{S}_{cc}(u) - S_0(t) \big )du$ converges to a Wiener process and the integration of a Wiener process is a Wiener process, it follows that $\int _0^\tau \sqrt{N}\big (\hat{S}_{cc}(u) - S_0(t) \big )du$ converges to a normal variable with mean zero and variance $\int _0^\tau \overline{\mu }^2(\tau ,u) d\textrm{Var}_s(u)$ where $\overline{\mu }(\tau ,u) = \mu (\tau ) - \mu (u) = \int _u^\tau S(v)dv$ and $\textrm{Var}_s(u) = \int _0^u \pi ^{-1}(v)d\varLambda _0(v) + (\sum _{b=1}^B p_b \frac{1-\alpha _b}{\alpha _b})\textrm{E}\big [\int _0^u Y(v)(1-\varDelta )\pi ^{-1}(v)d\varLambda _0(v) \big ]^2$.$\square$

### Appendix 1.3: Proof of Corollary 1

#### Proof

Using the result of Theorem 1, within each propensity score stratum, $\hat{\mu }_{k1}(\tau ) = \int _0^\tau \hat{S}_{cc,k1}(t)dt$ is asymptotically normal with mean $\mu ^1_k(\tau )$ and variance $\textrm{Var}(\hat{\mu }_{k1}(\tau ))$. Similarly, $\hat{\mu }_{k0}(\tau )= \int _0^\tau \hat{S}_{cc,k0}(t)dt$ is also asymptotically normal with mean $\mu ^0_k(\tau )$ and variance $\textrm{Var}(\hat{\mu }_{k0}(\tau ))$. Given that $\hat{\mu }_{k1}(\tau )$ and $\hat{\mu }_{k0}(\tau )$ are independent, $\hat{\nu }_k(\tau ) = \hat{\mu }_{k1}(\tau ) - \hat{\mu }_{k0}(\tau )$ is also asymptotically normal with mean $\nu _k(\tau )$ and variance $\textrm{Var}(\hat{\mu }_{k1}(\tau )) + \textrm{Var}(\hat{\mu }_{k0}(\tau ))$. Moreover, $\hat{\nu }_k(\tau )$ ($k=1,...,K$) are independent of each other and $w_k$ converges to $\textrm{E}(w_k)$ in probability as $n_k$ goes to infinity. Therefore, $\hat{\nu }(\tau ) = \sum _{k=1}^K w_k \hat{\nu }_k(\tau )$ is asymptotically normal with mean $\nu (\tau )=\sum _{k=1}^K \textrm{E}(w_k) \nu _k(\tau )$ and variance $\textrm{Var}(\hat{\nu }) = \sum _{k=1}^K [\textrm{E}(w_k)]^2 \big [\textrm{Var}(\hat{\mu }_{k1}(\tau )) + \textrm{Var}(\hat{\mu }_{k0}(\tau ))\big ]$.$\square$

### Appendix 1.4: Simulation results on ATT estimation

See Tables 5 and 6.

**Table 5:** Performance of estimation methods for marginal RMST treatment effect for the treated (ATT) in the scenario where treatment is observed for the full cohort. Est. TE, ESE, and SE are multiplied by 1,000 for ease of presentation. Full Cohort estimates causal effect using full-cohort data without confounding adjustment. Full Cohort PS uses full-cohort data with confounding adjustment. CC uses case-cohort data with confounding adjustment. MI uses multiple-imputation to impute the case-cohort data with confounding adjustment. Mis-MI uses a misspecified multiple-imputation model to impute the case-cohort data with confounding adjustment

N	R	Method	Est.	Abs. bias	ESE	SE	CP
			$\beta _A = 0$, $\varDelta _{ATE}=0$				
3000	1	Full Cohort	1.24	1.24	0.61	0.61	0.49
3000	1	Full Cohort PS	0.07	0.07	0.59	0.64	0.97
3000	1	CC	0.22	0.22	0.71	0.65	0.92
3000	1	MI	0.09	0.09	0.58	0.66	0.98
3000	1	Mis MI	0.17	0.17	0.60	0.68	0.98
3000	2	Full Cohort	1.24	1.24	0.61	0.61	0.49
3000	2	Full Cohort PS	0.07	0.07	0.59	0.64	0.97
3000	2	CC	0.10	0.10	0.60	0.59	0.96
3000	2	MI	0.08	0.08	0.58	0.65	0.97
3000	2	Mis MI	0.15	0.15	0.60	0.67	0.97
10000	1	Full Cohort	1.25	1.25	0.33	0.33	0.03
10000	1	Full Cohort PS	0.09	0.09	0.31	0.33	0.96
10000	1	CC	0.13	0.13	0.35	0.33	0.93
10000	1	MI	0.09	0.09	0.31	0.34	0.96
10000	1	Mis MI	0.19	0.19	0.33	0.35	0.95
10000	2	Full Cohort	1.25	1.25	0.33	0.33	0.03
10000	2	Full Cohort PS	0.09	0.09	0.31	0.33	0.96
10000	2	CC	0.11	0.11	0.33	0.32	0.94
10000	2	MI	0.09	0.09	0.31	0.33	0.96
10000	2	Mis MI	0.17	0.17	0.33	0.34	0.95

N	R	Method	Est.	% bias	ESE	SE	CP
			$$\beta _A=-2.5,$$$$\varDelta _{ATE}=17.76$$				
3000	1	Full Cohort	21.91	40.36	2.00	1.93	0.09
3000	1	Full Cohort PS	16.13	3.38	1.98	2.09	0.96
3000	1	CC	17.06	9.29	2.49	2.26	0.91
3000	1	MI	16.32	4.54	1.99	2.20	0.98
3000	1	Mis MI	16.76	7.39	2.05	2.29	0.96
3000	2	Full Cohort	21.91	40.36	2.00	1.93	0.09
3000	2	Full Cohort PS	16.13	3.38	1.98	2.09	0.96
3000	2	CC	16.43	5.27	2.11	1.99	0.93
3000	2	MI	16.21	3.85	2.00	2.14	0.96
3000	2	Mis MI	16.62	6.49	2.06	2.21	0.95
10000	1	Full Cohort	21.81	39.72	0.95	1.05	0.00
10000	1	Full Cohort PS	15.98	2.37	0.97	1.06	0.96
10000	1	CC	16.17	3.61	1.20	1.14	0.91
10000	1	MI	16.09	3.10	0.99	1.10	0.96
10000	1	Mis MI	16.58	6.26	1.06	1.15	0.91
10000	2	Full Cohort	21.81	39.72	0.95	1.05	0.00
10000	2	Full Cohort PS	15.98	2.37	0.97	1.06	0.96
10000	2	CC	16.06	2.92	1.06	1.07	0.94
10000	2	MI	16.02	2.63	0.98	1.08	0.96
10000	2	Mis MI	16.45	5.41	1.04	1.12	0.91

**Table 6:** Performance of estimation methods for the marginal RMST treatment effect for the treated (ATT) under the scenario where treatment is only observed for the case-cohort. Est. TE, bias, ESE, and SE are multiplied by 1,000 for ease of presentation. Full Cohort estimates causal effect using full-cohort data without confounding adjustment. Full Cohort PS uses full-cohort data with confounding adjustment. CC uses case-cohort data with confounding adjustment. MI uses multiple-imputation to impute the case-cohort data with confounding adjustment. Mis-MI uses a misspecified multiple-imputation model to impute the case-cohort data with confounding adjustment

N	R	Method	Est.	Abs. bias	ESE	SE	CP
			$\beta _A = 0$, $\varDelta _{ATE}=0$				
3000	1	Full Cohort	1.24	1.24	0.61	0.61	0.49
3000	1	Full Cohort PS	0.07	0.07	0.59	0.64	0.97
3000	1	CC	0.23	0.23	0.67	0.65	0.93
3000	1	MI	0.43	0.43	0.64	0.73	0.96
3000	1	Mis MI	0.50	0.50	0.66	0.75	0.96
3000	2	Full Cohort	1.24	1.24	0.61	0.61	0.49
3000	2	Full Cohort PS	0.07	0.07	0.59	0.64	0.97
3000	2	CC	0.15	0.15	0.63	0.60	0.94
3000	2	MI	0.22	0.22	0.60	0.68	0.97
3000	2	Mis MI	0.28	0.28	0.61	0.70	0.97
10000	1	Full Cohort	1.25	1.25	0.33	0.33	0.03
10000	1	Full Cohort PS	0.09	0.09	0.31	0.33	0.96
10000	1	CC	0.12	0.12	0.33	0.34	0.96
10000	1	MI	0.41	0.41	0.35	0.37	0.85
10000	1	Mis MI	0.48	0.48	0.36	0.38	0.79
10000	2	Full Cohort	1.25	1.25	0.33	0.33	0.03
10000	2	Full Cohort PS	0.09	0.09	0.32	0.32	0.94
10000	2	CC	0.11	0.11	0.33	0.32	0.94
10000	2	MI	0.24	0.24	0.33	0.35	0.94
10000	2	Mis MI	0.30	0.30	0.34	0.36	0.90

N	R	Method	Est.	% bias	ESE	SE	CP
			$$\beta _A=-2.5,$$$$\varDelta _{ATE}=17.76$$				
3000	1	Full Cohort	21.91	40.36	2.00	1.93	0.09
3000	1	Full Cohort PS	16.13	3.38	1.98	2.09	0.96
3000	1	CC	17.02	8.97	2.48	2.26	0.91
3000	1	MI	18.50	18.52	2.32	2.62	0.86
3000	1	Mis MI	18.99	21.64	2.41	2.70	0.82
3000	2	Full Cohort	21.91	40.36	2.00	1.93	0.09
3000	2	Full Cohort PS	16.13	3.38	1.98	2.09	0.96
3000	2	CC	16.39	4.94	2.16	1.99	0.93
3000	2	MI	17.15	9.88	2.07	2.32	0.95
3000	2	Mis MI	17.48	12.03	2.13	2.38	0.93
10000	1	Full Cohort	21.81	39.72	0.95	1.05	0.00
10000	1	Full Cohort PS	15.98	2.37	0.97	1.06	0.96
10000	1	CC	16.20	3.76	1.15	1.15	0.93
10000	1	MI	18.10	15.98	1.23	1.32	0.55
10000	1	Mis MI	18.64	19.45	1.27	1.37	0.38
10000	2	Full Cohort	21.81	39.72	0.95	1.05	0.00
10000	2	Full Cohort PS	15.98	2.37	0.97	1.06	0.96
10000	2	CC	16.05	2.79	1.04	1.07	0.95
10000	2	MI	16.88	8.14	1.06	1.17	0.83
10000	2	Mis MI	17.26	10.58	1.10	1.20	0.77

### Appendix 1.5: Simulation Study with 90$\%$ Censoring Rate

See Figs. 3 and 4; Tables 7 and 8.

**Table 7:** Performance of estimation methods for the marginal RMST treatment effect (ATE) in the scenario where treatment is observed only for the case-cohort and $95\%$ censoring rate. Est. TE, ESE, and SE are multiplied by 1,000 for ease of presentation. Full Cohort estimates causal effect using full-cohort data without confounding adjustment. Full Cohort PS uses full-cohort data with confounding adjustment. CC uses case-cohort data with confounding adjustment. MI uses multiple-imputation to impute the case-cohort data with confounding adjustment. Mis-MI uses a misspecified multiple-imputation model to impute the case-cohort data with confounding adjustment

N	R	Method	Est.	Abs. bias	ESE	SE	CP
			$\beta _A = 0$, $\varDelta _{ATE} = 0$				
40000	1	Full Cohort	0.08	0.08	0.02	0.02	0.03
40000	1	Full Cohort PS	0.01	0.01	0.02	0.02	0.93
40000	1	CC	0.01	0.01	0.02	0.02	0.90
40000	1	MI	0.04	0.04	0.02	0.02	0.59
40000	1	Mis MI	0.04	0.04	0.02	0.02	0.57
40000	2	Full Cohort	0.08	0.08	0.02	0.02	0.03
40000	2	Full Cohort PS	0.01	0.01	0.02	0.02	0.93
40000	2	CC	0.01	0.01	0.02	0.02	0.91
40000	2	MI	0.03	0.03	0.02	0.02	0.78
40000	2	Mis MI	0.03	0.03	0.02	0.02	0.76
60000	1	Full Cohort	0.08	0.08	0.02	0.02	0.00
60000	1	Full Cohort PS	0.01	0.01	0.02	0.02	0.91
60000	1	CC	0.01	0.01	0.02	0.02	0.90
60000	1	MI	0.04	0.04	0.02	0.02	0.45
60000	1	Mis MI	0.04	0.04	0.02	0.02	0.39
60000	2	Full Cohort	0.08	0.08	0.02	0.02	0.00
60000	2	Full Cohort PS	0.01	0.01	0.02	0.02	0.91
60000	2	CC	0.01	0.01	0.02	0.02	0.91
60000	2	MI	0.02	0.02	0.02	0.02	0.73
60000	2	Mis MI	0.03	0.03	0.02	0.02	0.70

N	R	Method	Est.	% bias	ESE	SE	CP
			$\beta _A = -2.5$, $\varDelta _{ATE} = 0.91$				
40000	1	Full Cohort	1.14	26.13	0.06	0.06	0.02
40000	1	Full Cohort PS	0.92	1.65	0.05	0.05	0.97
40000	1	CC	0.93	2.22	0.06	0.06	0.95
40000	1	MI	1.03	13.86	0.06	0.06	0.45
40000	1	Mis MI	1.05	15.56	0.06	0.06	0.38
40000	2	Full Cohort	1.14	26.13	0.06	0.06	0.02
40000	2	Full Cohort PS	0.92	1.65	0.05	0.05	0.97
40000	2	CC	0.92	1.87	0.05	0.05	0.96
40000	2	MI	0.98	8.08	0.05	0.06	0.80
40000	2	Mis MI	0.99	9.28	0.05	0.06	0.73
60000	1	Full Cohort	1.15	26.83	0.05	0.05	0.00
60000	1	Full Cohort PS	0.93	2.25	0.04	0.04	0.93
60000	1	CC	0.93	2.58	0.05	0.05	0.91
60000	1	MI	1.04	14.37	0.05	0.05	0.25
60000	1	Mis MI	1.05	16.08	0.05	0.05	0.18
60000	2	Full Cohort	1.15	26.83	0.05	0.05	0.00
60000	2	Full Cohort PS	0.93	2.25	0.04	0.04	0.93
60000	2	CC	0.93	2.35	0.05	0.04	0.93
60000	2	MI	0.98	8.61	0.05	0.05	0.63
60000	2	Mis MI	0.99	9.81	0.05	0.05	0.55

**Table 8:** Performance of estimation methods for the marginal RMST treatment effect for the treated (ATT) under the scenario where treatment is observed only for the case-cohort and $95\%$ censoring rate. Est. TE, ESE, and SE are multiplied by 1,000 for ease of presentation. Full Cohort estimates causal effect using full-cohort data without confounding adjustment. Full Cohort PS uses full-cohort data with confounding adjustment. CC uses case-cohort data with confounding adjustment. MI uses multiple-imputation to impute the case-cohort data with confounding adjustment. Mis-MI uses a misspecified multiple-imputation model to impute the case-cohort data with confounding adjustment

N	R	Method	Est.	Abs. bias	ESE	SE	CP
			$\beta _A = 0$, $\varDelta _{ATE} = 0$				
40000	1	Full Cohort	0.08	0.08	0.02	0.02	0.03
40000	1	Full Cohort PS	0.01	0.01	0.02	0.02	0.95
40000	1	CC	0.01	0.01	0.02	0.02	0.93
40000	1	MI	0.03	0.03	0.02	0.02	0.71
40000	1	Mis MI	0.04	0.04	0.02	0.02	0.66
40000	2	Full Cohort	0.08	0.08	0.02	0.02	0.03
40000	2	Full Cohort PS	0.01	0.01	0.02	0.02	0.95
40000	2	CC	0.01	0.01	0.02	0.02	0.93
40000	2	MI	0.02	0.02	0.02	0.02	0.85
40000	2	Mis MI	0.02	0.02	0.02	0.02	0.81
60000	1	Full Cohort	0.08	0.08	0.02	0.02	0.00
60000	1	Full Cohort PS	0.01	0.01	0.02	0.02	0.93
60000	1	CC	0.01	0.01	0.02	0.02	0.91
60000	1	MI	0.03	0.03	0.02	0.02	0.58
60000	1	Mis MI	0.04	0.04	0.02	0.02	0.49
60000	2	Full Cohort	0.08	0.08	0.02	0.02	0.00
60000	2	Full Cohort PS	0.01	0.01	0.02	0.02	0.93
60000	2	CC	0.01	0.01	0.02	0.02	0.91
60000	2	MI	0.02	0.02	0.02	0.02	0.79
60000	2	Mis MI	0.02	0.02	0.02	0.02	0.75

N	R	Method	Est.	% bias	ESE	SE	CP
			$\beta _A = -2.5$, $\varDelta _{ATE} = 0.77$				
40000	1	Full Cohort	1.14	48.26	0.06	0.06	0.00
40000	1	Full Cohort PS	0.79	2.91	0.05	0.06	0.96
40000	1	CC	0.80	3.75	0.06	0.06	0.94
40000	1	MI	0.91	17.70	0.06	0.07	0.40
40000	1	Mis MI	0.93	20.52	0.06	0.07	0.31
40000	2	Full Cohort	1.14	48.26	0.06	0.06	0.00
40000	2	Full Cohort PS	0.79	2.91	0.05	0.06	0.96
40000	2	CC	0.80	3.18	0.05	0.05	0.95
40000	2	MI	0.85	10.65	0.05	0.06	0.77
40000	2	Mis MI	0.87	12.69	0.06	0.06	0.68
60000	1	Full Cohort	1.15	49.07	0.05	0.05	0.00
60000	1	Full Cohort PS	0.80	3.55	0.04	0.04	0.92
60000	1	CC	0.80	4.01	0.05	0.05	0.89
60000	1	MI	0.91	18.19	0.05	0.05	0.19
60000	1	Mis MI	0.93	21.06	0.05	0.05	0.12
60000	2	Full Cohort	1.15	49.07	0.05	0.05	0.00
60000	2	Full Cohort PS	0.80	3.55	0.04	0.04	0.92
60000	2	CC	0.80	3.71	0.04	0.04	0.90
60000	2	MI	0.86	11.22	0.05	0.05	0.59
60000	2	Mis MI	0.87	13.27	0.05	0.05	0.47

### Appendix 1.6: Simulation study examining the effect of *K* PS strata on treatment effect estimation

Table 9 shows how the number of PS strata ($K=$ 5, 7, and 10) affects the performance of the CC method. The simulation setup is the same as the main text. For smaller sample sizes ($N=4000$), estimate bias will increase when *K* is too large because the number of cases per stratum is small. When the sample size increases, the number of cases per stratum also increases, allowing for better effect estimation. When $K\ge 7$, we do not see much reduction in bias as K increases. Thus, $K=7$ appears to be a sensible choice.

**Table 9:** Performance of CC method for the marginal RMST treatment effect (ATE) under the scenario where treatment is only observed for the case-cohort. The number of PS strata are $K=$5, 7, 10. $\varDelta _{ATE} = 0.906$ and $\beta _A = -2.5$

N	R	Est effect (K=5)	Est effect (K=7)	Est effect (K=10)	% bias (K=5)	% bias (K=7)	% bias (K=10)
4000	1	0.95	0.95	0.96	4.96	4.91	6.30
4000	2	0.94	0.93	0.93	3.32	2.74	2.72
8000	1	0.95	0.95	0.95	4.98	4.42	4.37
8000	2	0.94	0.94	0.93	4.09	3.34	2.78
10000	1	0.95	0.94	0.94	4.55	3.81	3.69
10000	2	0.94	0.93	0.93	3.76	2.90	2.25

### Appendix 1.7: Simulation study of case-cohort design without stratification

See Figs. 5 and 6.
